# Contained Perforation of a Jejunal Diverticulum in an Elderly Patient: A Case Report

**DOI:** 10.7759/cureus.87719

**Published:** 2025-07-11

**Authors:** Antonio de Jesús González Luna, Uriel Covarrubias Robles, Christian Daniel Castrejón Cardona, Cristina Vanessa Cuevas Calla, Marco Antonio Castellanos López

**Affiliations:** 1 Department of General Surgery, Regional Hospital “Dr. Valentin Gomez Farias”, Institute of Security and Social Services for the State Workers (ISSSTE), Zapopan, MEX; 2 Department of General Surgery, National Polytechnic Institute, Mexico, MEX

**Keywords:** atypical presentation, contained perforation, jejunal diverticulosis, small bowel diverticula, small bowel surgery

## Abstract

Jejunal diverticulosis is an uncommon condition, and its complications-such as perforation-pose a diagnostic and therapeutic challenge, particularly in elderly patients. Contained perforation is especially difficult to identify due to its non-specific clinical presentation and subtle radiological findings.

We report the case of an 82-year-old woman with a medical history of atrial fibrillation, heart failure, chronic obstructive pulmonary disease (COPD), and irritable bowel syndrome. She presented to the emergency department with a two-day history of diffuse abdominal pain, nausea, vomiting, and diarrhea. Physical examination revealed abdominal distension and tenderness in the epigastric and mesogastric regions, without signs of peritoneal irritation. Laboratory tests showed leukocytosis and impaired renal and hepatic function. Although initial imaging studies suggested a possible bowel obstruction, a non-contrast-enhanced abdominopelvic computed tomography (CT) scan was diagnostic. It demonstrated multiple jejunal diverticula, one of which exhibited an elongated morphology, wall thinning, and adjacent mesenteric fat stranding-findings consistent with a contained perforation. Due to persistent symptoms despite conservative management, an exploratory laparotomy was performed. Intraoperatively, a perforated jejunal diverticulum was identified 90 cm distal to the ligament of Treitz. A 5 cm segmental small bowel resection was performed, followed by a stapled side-to-side anastomosis with a functionally end-to-end configuration. The postoperative course was uneventful, and the patient was discharged on postoperative day four with favorable outpatient follow-up.

This case underscores the importance of considering complicated jejunal diverticulitis in the differential diagnosis of acute abdominal pain in elderly patients, even in the presence of atypical clinical findings. A non-contrast CT scan was essential for the early identification of the contained perforation, allowing for timely surgical intervention and a favorable outcome. A high index of clinical suspicion is crucial in this patient population to avoid potentially fatal diagnostic delays.

## Introduction

Jejunal diverticulosis is an uncommon clinical entity, with a reported prevalence ranging from 0.06% to 1.3% [1]. These jejunal diverticula are false diverticula, consisting of herniation of the mucosa and submucosa through the muscularis propria, and are asymptomatic in most cases. However, up to 40% of patients may develop complications such as chronic abdominal pain, malabsorption, gastrointestinal bleeding, or acute events including diverticulitis, obstruction, or perforation [2,3]. Perforation, which occurs in approximately 6% of cases, is the most severe complication and carries an estimated mortality rate of 20% to 30% [1,4].

The diagnosis of acute jejunal diverticulitis is clinically challenging, particularly in elderly patients with multiple comorbidities, due to its non-specific presentation. Although contrast-enhanced computed tomography (CT) is the imaging modality of choice for identifying complications, its findings may be subtle, leading to diagnostic delays. Isolated perforation of a jejunal diverticulum is extremely rare [4]. The treatment of choice in such cases is surgical resection of the affected segment with primary anastomosis, which remains the most effective therapeutic strategy to prevent adverse outcomes [2,4]. In this context, we present the case of a patient with a contained perforation of a jejunal diverticulum, diagnosed by CT and successfully treated with surgery.

## Case presentation

An 82-year-old female patient with a medical history of atrial fibrillation, heart failure, chronic obstructive pulmonary disease (COPD), and irritable bowel syndrome (IBS) presented to the emergency department with a 48-hour history of diffuse abdominal pain, predominantly in the mesogastrium, described as pressure-like in nature. The pain was associated with abdominal distension, nausea, and two episodes of non-bilious vomiting. She also reported intermittent diarrhea, which, while not a classic manifestation of intestinal perforation, may occur in specific clinical contexts. In this case, her prior diagnosis of IBS could partially account for this symptom. Furthermore, limited contamination or localized peritoneal irritation in cases of contained perforation may trigger changes in bowel habits, including intermittent diarrhea. Her surgical history included laparoscopic cholecystectomy and bilateral tension-free inguinal hernioplasty.

On physical examination, the patient was hemodynamically stable, with signs of dehydration evident on the oral mucosa. Her abdomen was markedly distended, with increased bowel sounds and tympanic percussion. Deep palpation revealed moderate tenderness in the epigastric and mesogastric regions without signs of peritoneal irritation.

**Figure 1:**
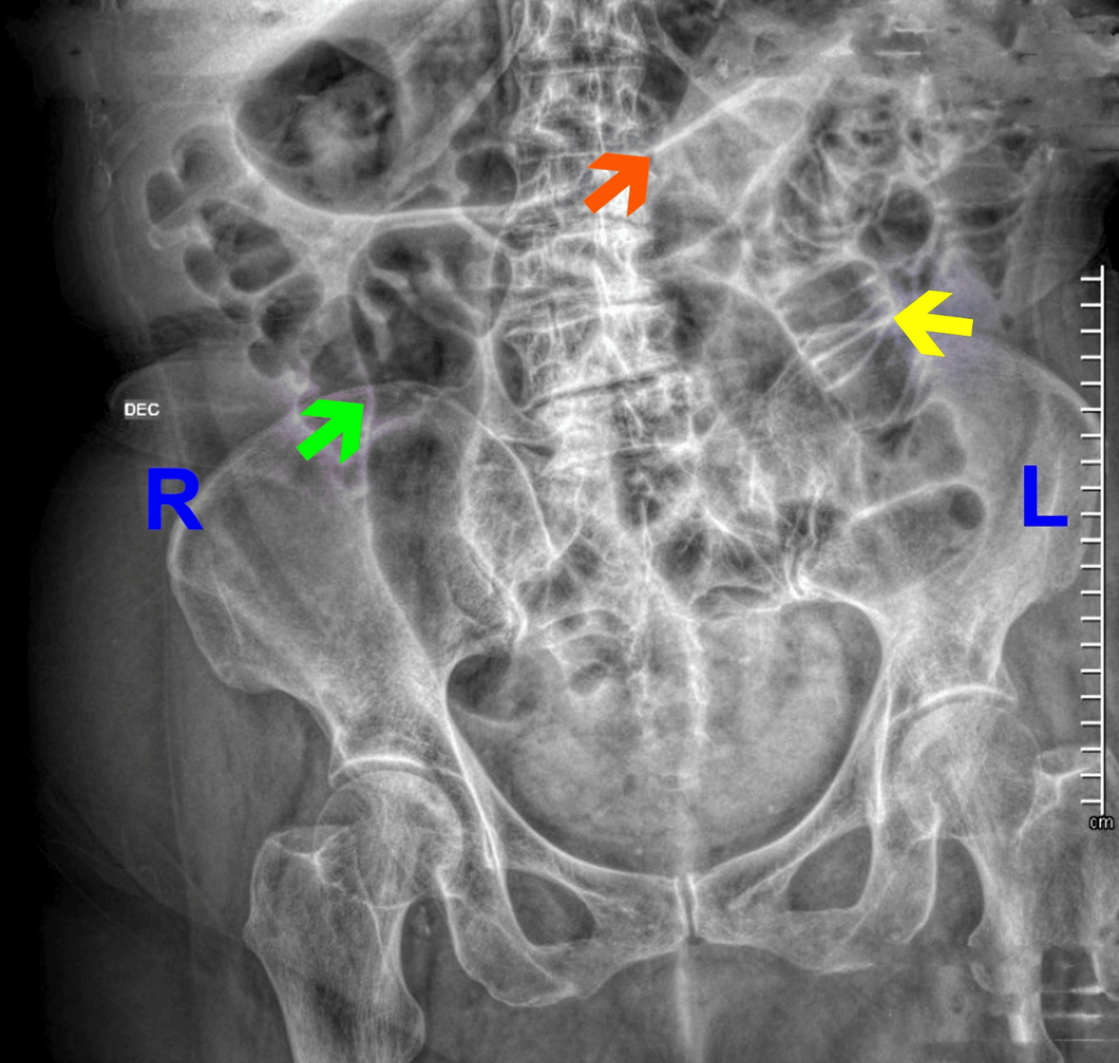
Supine abdominal radiograph. The image demonstrates small bowel distention (green arrow), interloop edema (orange arrow), and the "stacked coin" sign (yellow arrow), findings consistent with small bowel obstruction.

**Figure 2:**
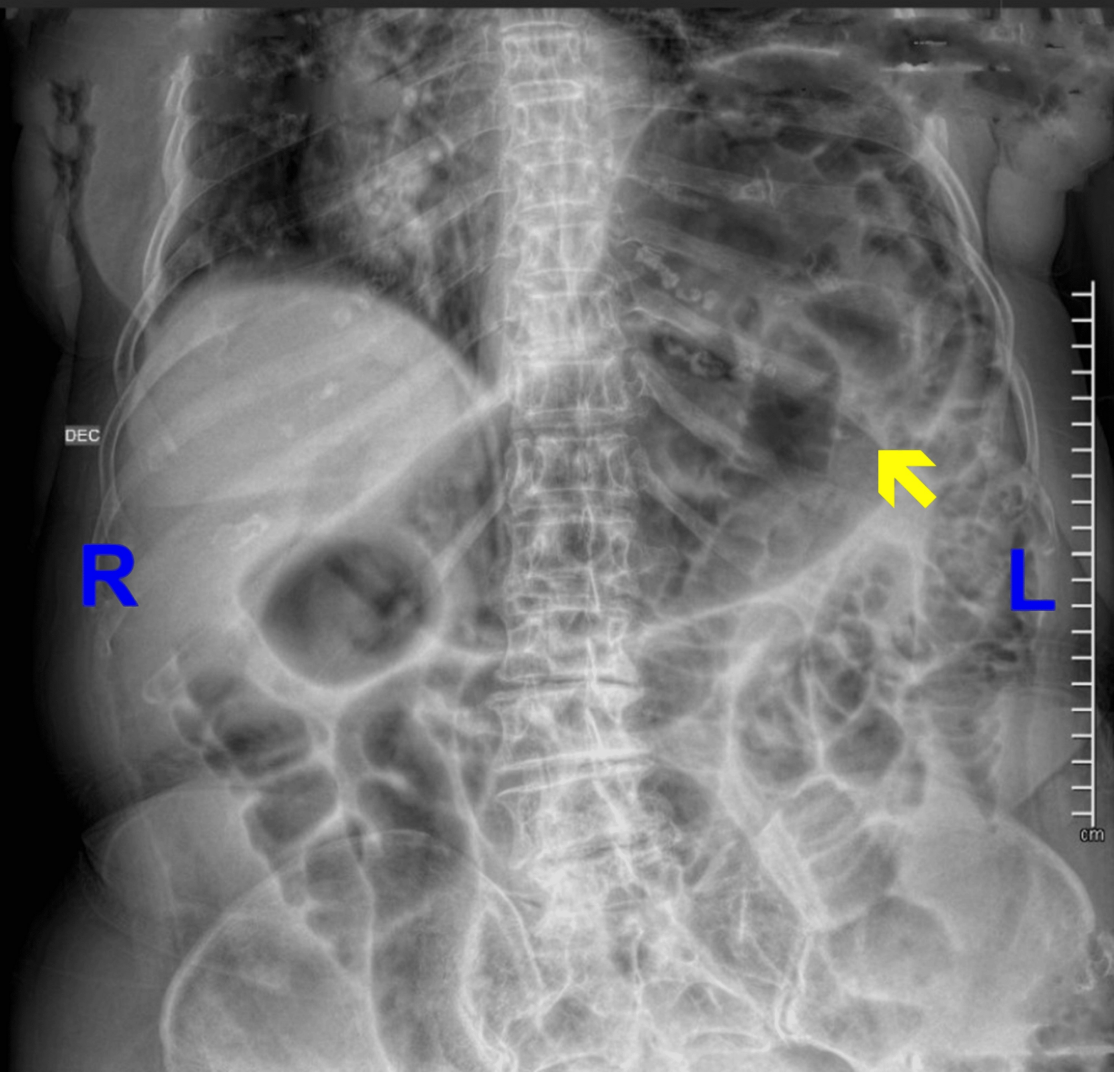
Supine abdominal radiograph. The image shows gastric distension (yellow arrow). No subdiaphragmatic free air is observed.

Initial management included nasogastric decompression, intravenous hydration, and analgesia. A general surgery consultation was obtained. Due to the persistence of symptoms, imaging studies were requested. A supine abdominal radiograph revealed dilated small bowel loops with interloop edema and a characteristic “stacked coins” appearance (Figure 1). A chest radiograph showed no evidence of free subdiaphragmatic air and revealed stomach distension (Figure 2).

**Figure 3:**
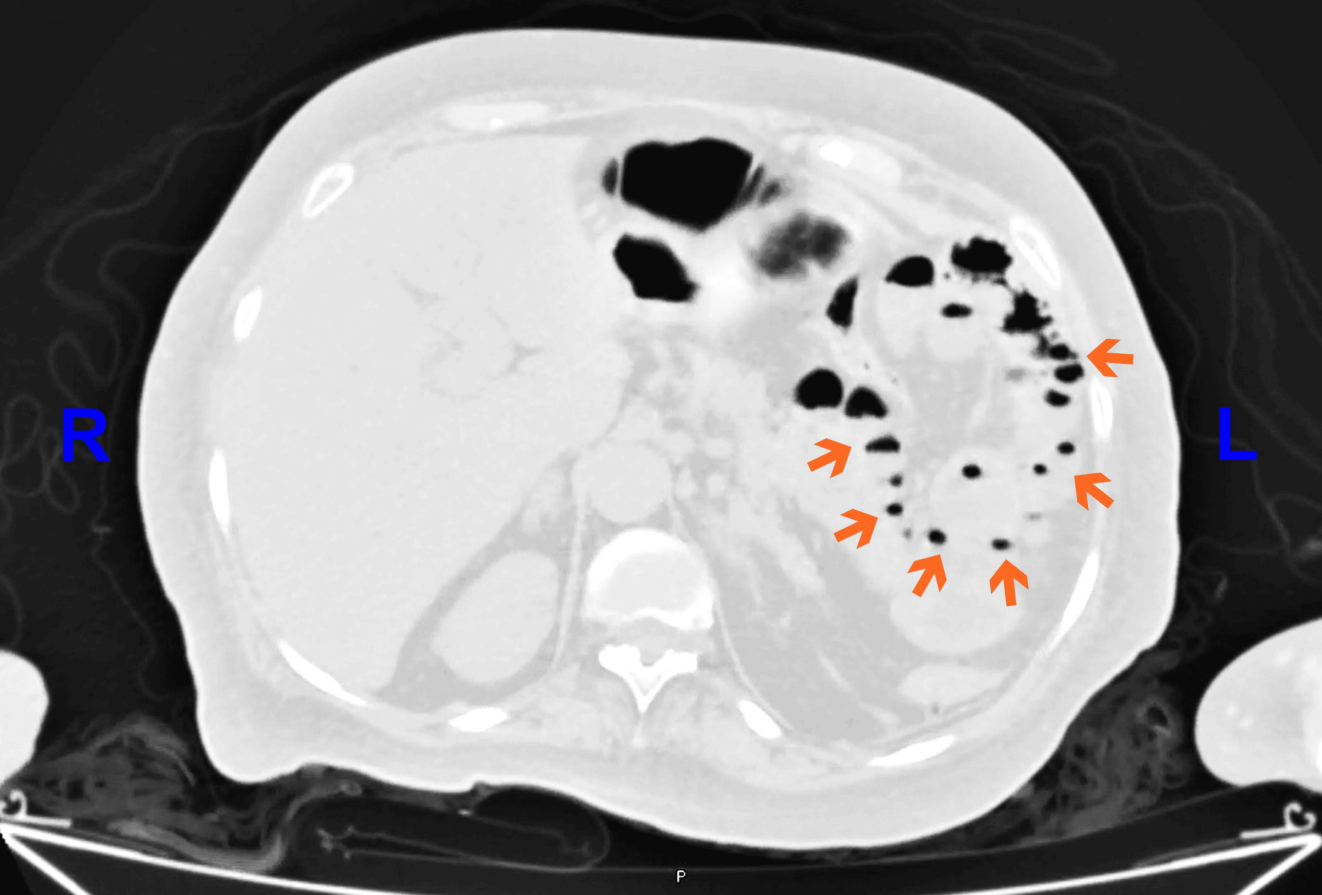
Axial non-contrast abdominopelvic computed tomography (CT) in lung window. Multiple sac-like protrusions (orange arrows) are seen arising from the mesenteric border of the jejunal wall in the lower left quadrant, consistent with jejunal diverticula. These outpouchings contain air or air-fluid levels. The adjacent bowel wall is mildly and symmetrically thickened. No evidence of pneumoperitoneum.

**Figure 4:**
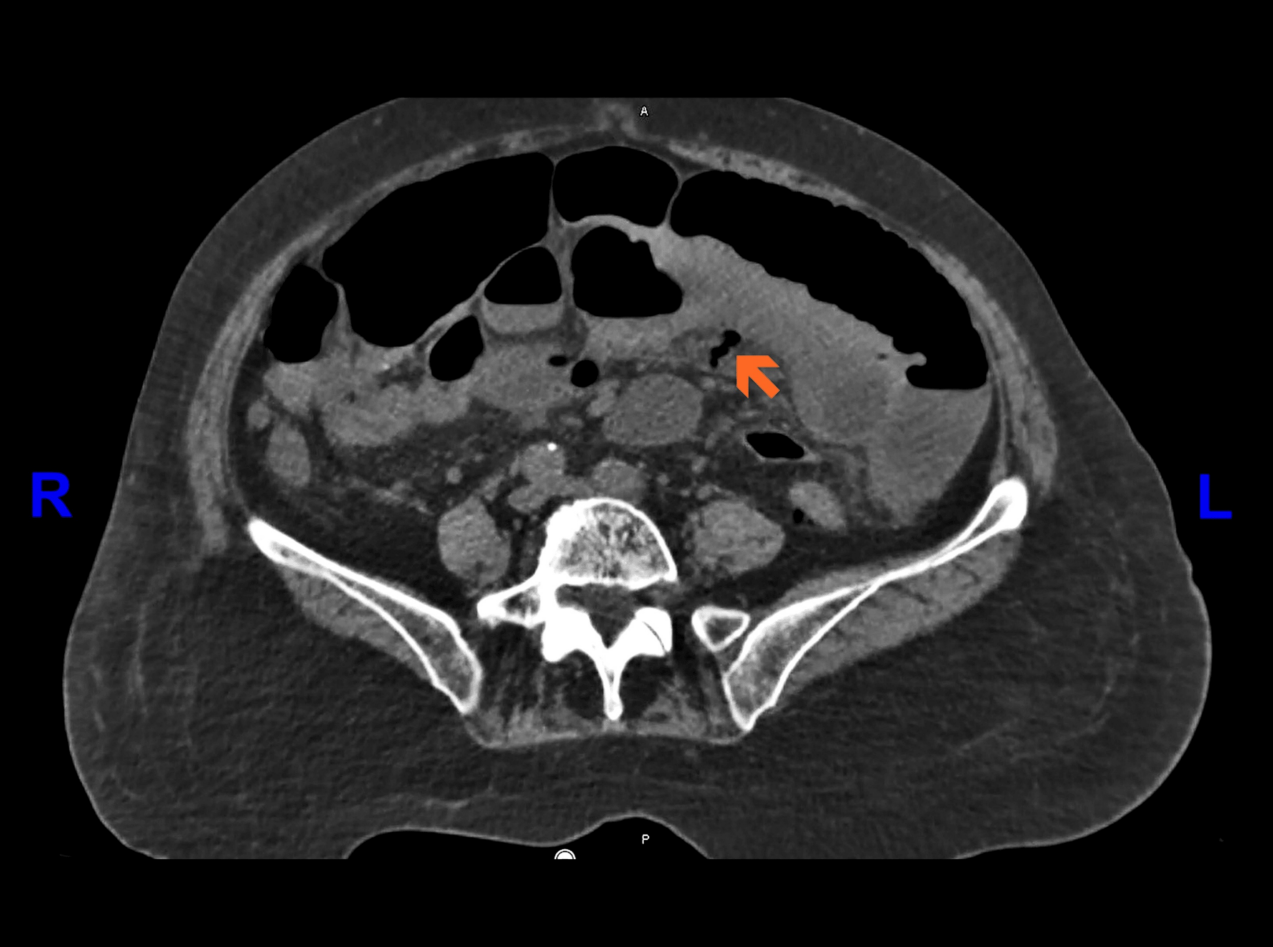
Non-contrast axial abdominopelvic computed tomography (CT) scan. A well-defined, elongated sac-like diverticulum (orange arrow) arising from a jejunal loop is observed in the central to left lower abdominal quadrant. The diverticulum contains air and lacks a clearly delineated wall along its course. Perilesional fat stranding and mesenteric thickening are noted, suggesting localized inflammatory changes. No free fluid or extraluminal air is present in this slice.

Given the non-specific findings, an abdominopelvic CT scan without intravenous contrast was performed. This decision was made due to the unavailability of intravenous contrast material at the time of imaging in our facility. The scan demonstrated multiple sacculations along the mesenteric border of the jejunum, consistent with jejunal diverticulosis. Additionally, there was bowel wall thickening and surrounding mesenteric fat stranding (Figure 3). A prominent diverticulum with a finger-like morphology exhibited mural discontinuity and adjacent mesenteric stranding, findings highly suggestive of a contained perforation (Figure 4).

Laboratory tests at admission revealed mild leukocytosis 10.75 x 10³/μL (4.50-10.50), acute kidney injury: blood urea nitrogen 45.1 mg/dL (6.0-20.0), creatinine 1.61 mg/dL (0.50-0.90), and elevated transaminases: aspartate aminotransferase (AST) 154 U/L (10.0-36.0). Other parameters, including serum lactate 1.40 mmol/L (0.50-1.30), were within normal limits (Table 1).

**Table 1:** Laboratory results with reference ranges.

Parameter	Patient value	Reference range
Red blood cells (RBC)	3.71 × 10⁶/μL	4.20–5.40
Hemoglobin (Hb)	11.5 g/dL	12.6–16.6
Hematocrit (Hct)	35.1%	36.6–47.3
Platelets (PLT)	287 × 10³/μL	150–420
White blood cells (WBC)	10.75 × 10³/μL	4.50–10.50
Absolute neutrophil count (ANC)	9.19 × 10³/μL	2.50–7.00
Glucose	87 mg/dL	74–109
Blood urea nitrogen (BUN)	45.1 mg/dL	6.0–20.0
Creatinine	1.61 mg/dL	0.50–0.90
Total bilirubin	0.87 mg/dL	0.00–1.20
Direct bilirubin	0.48 mg/dL	0.00–0.20
Indirect bilirubin	0.39 mg/dL	0.30–0.80
Alanine aminotransferase (ALT)	15 U/L	10.0–35.0
Aspartate aminotransferase (AST)	154 U/L	10.0–36.0
Lactate dehydrogenase (LDH)	154 U/L	135–214
Calcium	8.6 mg/dL	8.4–10.2
Phosphorus	3.2 mg/dL	2.5–4.5
Chloride	102.4 mEq/L	98–107
Potassium	4.5 mEq/L	3.5–5.1
Sodium	135 mEq/L	136–145
Magnesium	2.0 mg/dL	1.6–2.3
Lactate	1.40 mmol/L	0.30–0.70

**Figure 5:**
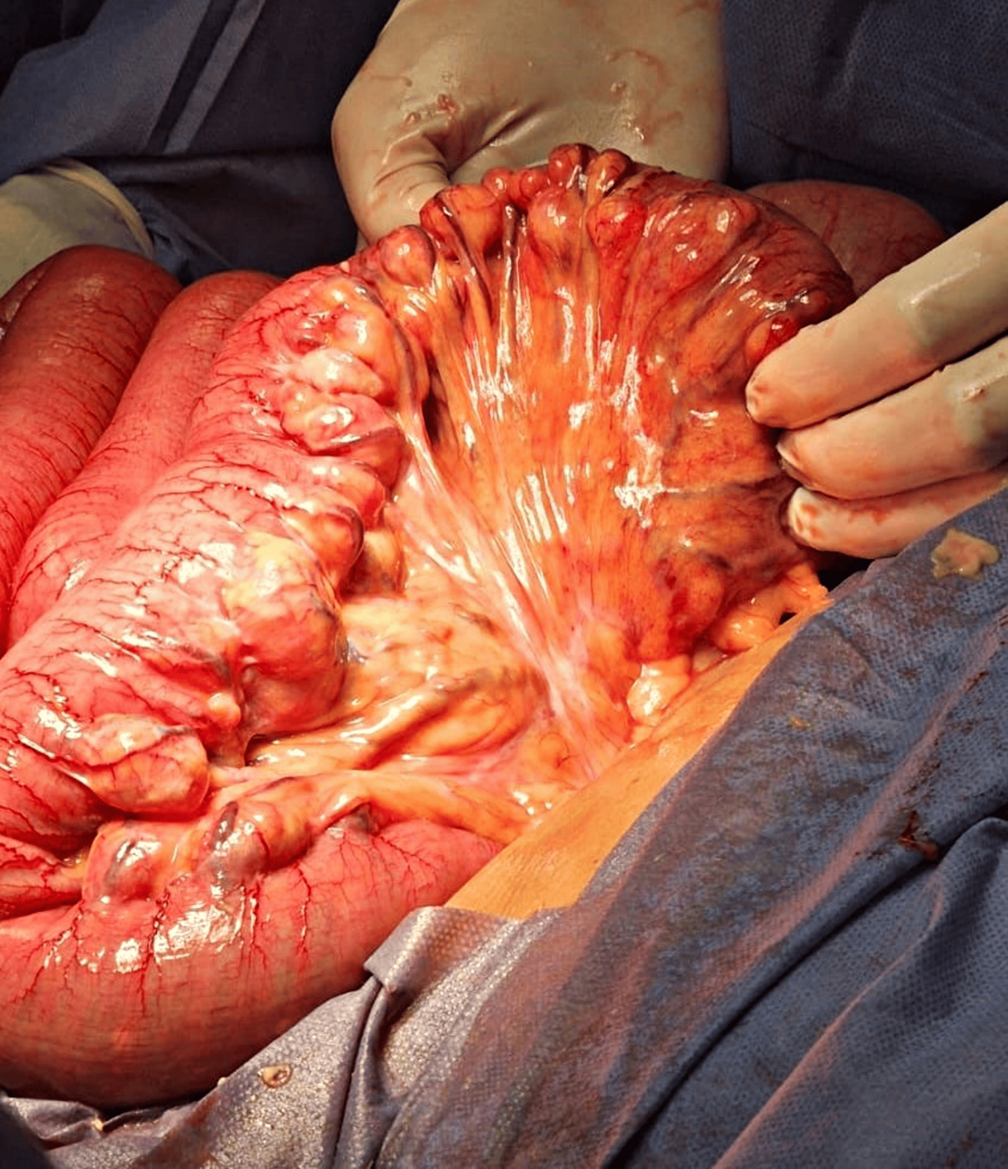
Jejunal diverticula. Intraoperative image showing multiple thin-walled jejunal diverticula located along the mesenteric border, with sac-like protrusions extending into the mesentery. No signs of active perforation are observed.

**Figure 6:**
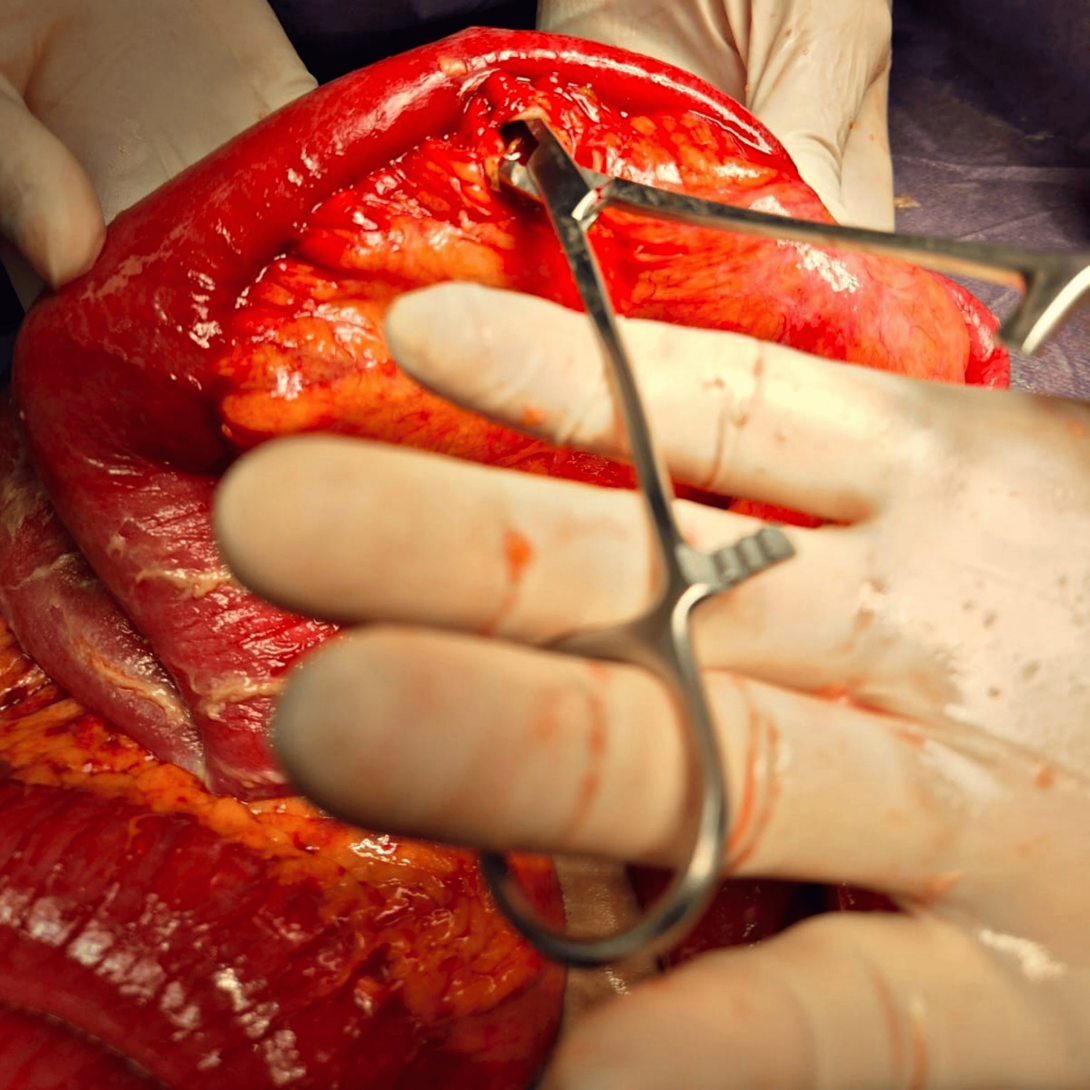
Perforated jejunal diverticulum. Intraoperative image showing a segment of jejunum with a perforation located on the antimesenteric border, approximately 90 cm distal to the ligament of Treitz. A dissecting forceps exposes the perforation orifice, which is surrounded by inflamed tissue and focal wall thickening.

Due to the suspicion of a contained perforation, an exploratory laparotomy was performed in the first hours after admission to the emergency department, via a supraumbilical midline incision, shortly after the abdominopelvic CT findings suggested this diagnosis. Intraoperative findings included marked small bowel distension. Systematic exploration from the ligament of Treitz revealed multiple jejunal diverticula. At 90 cm distal to this point, a perforated diverticulum sealed by adjacent mesentery was identified (Figures 5, 6).

**Figure 7:**
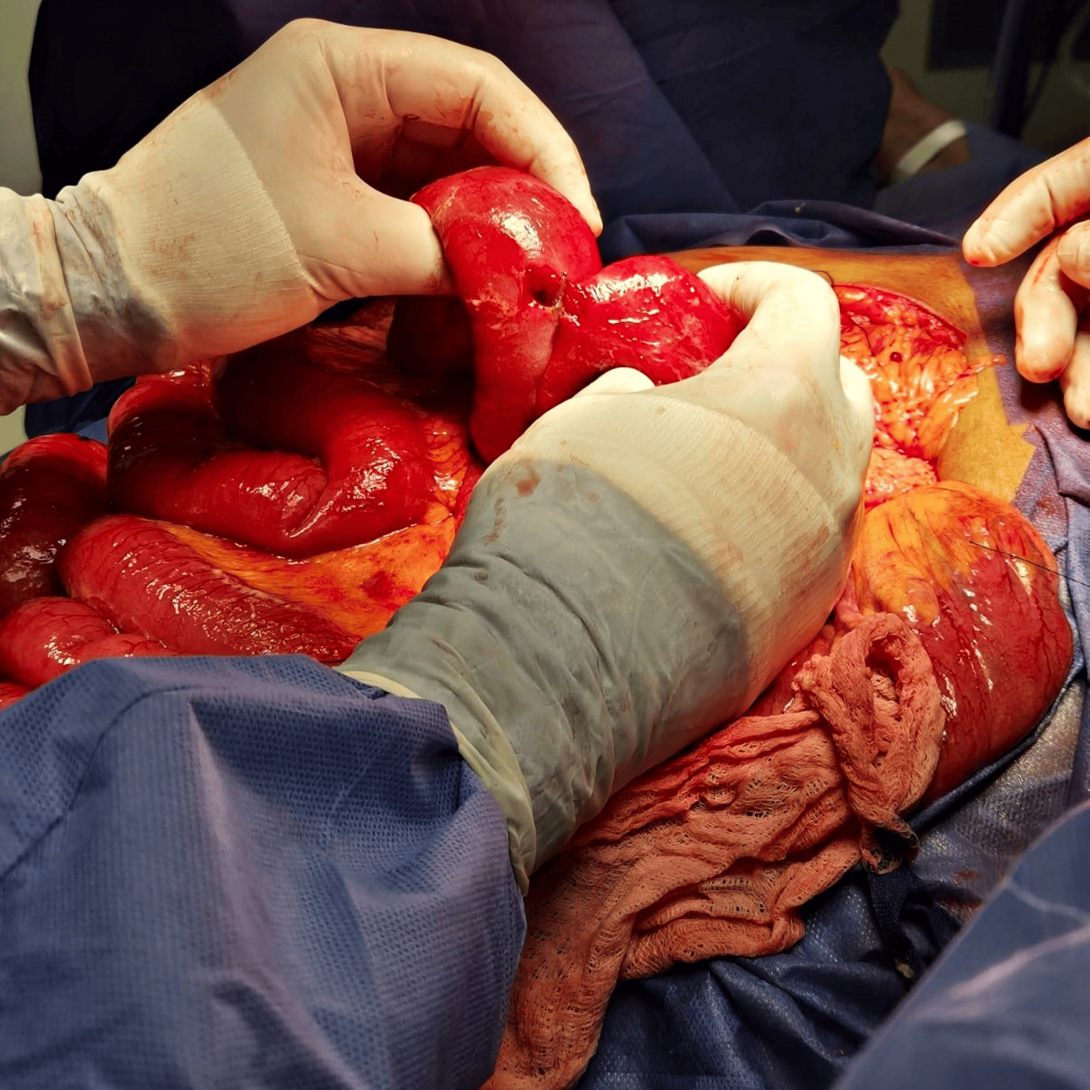
Anastomosis. A side-to-side enteroenteric anastomosis was performed using a mechanical stapling technique, achieving appropriate alignment and approximation of the bowel edges.

A 5 cm segmental resection of the affected jejunum was performed, followed by a side-to-side, functionally end-to-end, stapled jejunojejunostomy (Figure 7). The enterotomy was closed in two layers: an inner continuous Connell-Mayo suture with 2-0 PDS and an outer seromuscular Cushing suture using 3-0 silk. The mesenteric defect was closed with a continuous 3-0 silk suture. To ensure the integrity of the jejunojejunostomy and to rule out any additional perforated diverticula based on the clinical and intraoperative findings, both a methylene blue leak test and a pneumatic leak test were performed, with negative results.

The patient had an uneventful postoperative recovery. Oral intake was initiated at 24 hours and advanced without issues. She was discharged on postoperative day four with scheduled follow-up in the outpatient general surgery clinic.

## Discussion

This case report describes the successful management of a contained perforation of a jejunal diverticulum, a rare complication of an already uncommon condition [5,6]. The favorable outcome in this 82-year-old patient with multiple comorbidities was attributed to three key factors: a high index of clinical suspicion, timely imaging diagnosis despite technical limitations, and early surgical intervention.

The diagnosis of acute jejunal diverticulitis is particularly challenging due to its non-specific clinical presentation, often mimicking other causes of acute abdomen. While contrast-enhanced CT is considered the gold standard, with a sensitivity exceeding 90% [7], this case demonstrates that non-contrast CT can also be diagnostic. In this patient, the identification of a jejunal outpouching with mural discontinuity and surrounding mesenteric fat stranding provided highly suggestive indirect signs of contained perforation. These findings are consistent with those described by Tapias et al. [6], reinforcing the diagnostic value of non-contrast CT in scenarios where contrast administration is contraindicated or unavailable.

Once diagnostic suspicion is established, therapeutic decision-making becomes critical. Although conservative management has been described in highly selected cases of contained perforation [5], most of the available evidence supports surgical resection with primary anastomosis as the definitive treatment to prevent progression to peritonitis and sepsis. Literature reports a mortality rate of up to 40% in delayed cases, compared to approximately 5% when early resection is performed [8-10]. A high index of suspicion is essential in elderly patients presenting with non-specific abdominal pain and no pneumoperitoneum, as contained jejunal perforation must be considered [10].

Given the rarity of this condition, high-quality systematic reviews on the management of contained jejunal diverticular perforation are lacking. However, several narrative reviews and case series offer valuable clinical insight. Fleres et al. conducted an extensive review concluding that primary surgical resection remains the treatment of choice in complicated or perforated cases, whereas conservative management may be considered in hemodynamically stable patients who are carefully selected and exhibit no signs of sepsis or peritonitis [11]. Similarly, Leigh et al. and Baumgartner et al. presented case series and reports supporting early surgical intervention in most patients with contained perforation, particularly in older individuals or those with comorbidities [4,12].

In our patient, despite advanced age and comorbidities (COPD, atrial fibrillation, and heart failure), early surgery was successfully performed, demonstrating that major abdominal surgery remains a viable and safe option when supported by appropriate multidisciplinary planning and perioperative management [5,8].

The main limitations of this report include the lack of a long-term follow-up, which prevents the evaluation of late complications such as malabsorption syndrome, and the inherent limitations of a single case, which restrict generalizability. Nevertheless, several clinical recommendations emerge: (1) maintain a high index of suspicion for this condition in elderly patients presenting with atypical abdominal pain, (2) recognize the diagnostic utility of non-contrast CT when contrast-enhanced imaging is not feasible, and (3) prioritize early surgical resection even in cases of contained perforation to improve clinical outcomes. Although this is an isolated case, its clinical implications are significant for the timely diagnosis and management of a rare but potentially life-threatening condition.

## Conclusions

Complicated jejunal diverticulitis, although rare, should be included in the differential diagnosis of elderly patients presenting with acute abdominal pain. This case demonstrates that timely diagnosis is achievable even with non-contrast CT, provided that indirect signs of contained perforation are accurately recognized. Early surgical intervention, consisting of resection and primary anastomosis, proved to be both safe and effective despite the patient's high operative risk, supporting the superiority of an active surgical approach over conservative management. This case highlights that a high index of clinical suspicion, expert radiologic interpretation, and prompt surgical decision-making are fundamental to achieving favorable outcomes.
